# Cytotoxic Effects of Pulp Capping Agents on Mesenchymal Stem Cells Isolated from Human Exfoliated Deciduous Teeth

**DOI:** 10.30476/dentjods.2023.99173.2131

**Published:** 2024-12-01

**Authors:** Bahareh Nazemi Salman, Ehsan Saburi, Mahtab Mohammadi Gheidari, Mahya Farsadeghi, Samira Basir Shabestari

**Affiliations:** 1 Dept. of Pediatrics, School of Dentistry, Zanjan University of Medical Sciences, Zanjan, Iran; 2 Dept. of Molecular Medicine, School of Medicine, Mashhad University of Medical Sciences, Mashhad, Iran; 3 Postgraduated Student of Endodontics, student research committee, Qazvin university of medical sciences,Qazvin, Iran; 4 General Dentist, School of Dentistry, Zanjan University of Medical Sciences, Zanjan, Iran; 5 ENT and Head and Neck Research Center and Department, The five senses Health Institute, Firoozgar Hospital, School of Medicine, Iran University of Medical Sciences, Tehran, Iran

**Keywords:** Dental pulp capping, Deciduous teeth, Stem Cells, Tooth

## Abstract

**Statement of the Problem:**

Success of pulpotomy of primary teeth depends on biological and cytotoxic effects of pulp capping agents. Mineral trioxide aggregate (MTA), Biodentine, calcium enriched mixture (CEM) cement, and ferric sulfate (FS) are among the commonly used pulp capping agents (PCAs) for pulpotomy, and their successful application has been previously evaluated.

**Purpose:**

This study aimed to compare the cytotoxicity of PCAs against mesenchymal stem cells isolated from human exfoliated deciduous teeth (SHEDs).

**Materials and Method:**

In this *in vitro* study, SHEDs were exposed to MTA, Biodentine, CEM cement, and FS for 24 and 72 hours. The methyl thiazolyl tetrazolium (MTT) assay was performed for five different concentrations of PCAs after 24 and 72 hours of exposure. Data were analyzed by ANOVA.

**Results:**

Generally, the biocompatibility increased by reduction in concentration. All tested concentrations showed higher biocompatibility at 72 hours compared with 24 hours (*p*< 0.0001). Comparison of cytotoxicity of different biomaterials revealed no significant difference at any time point (*p*> 0.05).

**Conclusion:**

In general, the cytotoxicity of MTA, Biodentine, CEM cement, and FS was comparable, with no significant difference. Cytotoxicity decreased over time and by a reduction in concentration of biomaterials. MTA and Biodentine showed maximum biocompatibility followed by FS, and CEM cement.

## Introduction

Preservation of pulp vitality until natural exfoliation of primary teeth is among the most important goals in pediatric dentistry [ 1 ]. Pulp therapy preserves the tooth vitality, enhances the function of primary teeth, and preserves the integrity and harmony of dental arch. Vital pulp therapy includes a range of conservative therapeutic procedures for resolution of pulpal inflammation, aiming to eliminate the inflammation and inflamed tissues, and preserving the radicular pulp vitality [ 2 ]. Pulpotomy is a type of vital pulp therapy, in which the coronal pulp is removed to allow the healthy radicular pulp to induce healing of the underlying pulp tissue by the effect of applied pulp capping agent in primary teeth [ 3 ]. Pulp capping agents (PCAs) serve as a protective barrier for the residual vital pulp tissue, and have optimal properties such as biocompatibility and bioactivity to induce the function of dental pulp stem cells (DPSCs) [ 4 ]. 

Stem cells (SCs) from human exfoliated deciduous teeth (SHEDs) or immature DPSCs are SCs originating from primary teeth, which are considered as a new population of SCs. They have a higher differentiation rate compared with mesenchymal SCs and DPSCs, and can differentiate into different cell lines such as neurons, odontogenic cells and adipocytes under *in vitro* conditions [ 5 ].

The methyl thiazolyl tetrazolium (MTT) assay measures the metabolic activity of the cells as a criterion for cell viability. This test is based on colorimetry, and conversion of yellow tetrazolium salt or MTT to purple formazan crystals by metabolically active cells. It is one of the standard assays for assessment of cytotoxicity of materials and cell proliferation by measuring the activity of the mitochondrial dehydrogenase enzyme [ 6 ].

Several studies have evaluated the response of DPSCs to different PCAs, and have proposed calcium hydroxide (CHO) as the most commonly used PCA for pulpotomy due to effective biological induction of SHEDs. Zinc oxide eugenol is also used for pulpotomy and pulpectomy due to eliciting a favorable pulpal response. Moreover, many studies have proposed mineral trioxide aggregate (MTA) as one of the most effective PCAs. Recently, novel biomaterials such as Biodentine, and calcium silicate-based cements were introduced with optimal biocompatibility and effective induction of SHEDs [ 7 - 8 ]. 

Biodentine is claimed to possess better physical and biological properties compared to other tricalcium silicate cements such as MTA and Bioaggregate TM (Bioaggregate) [ 9 ]. Recently, a new calcium silicate cement known as calcium enriched mixture (CEM) cement was introduced to the market with clinical properties similar to those of MTA, and chemical properties superior to those of MTA and Portland cement. CEM cement has antibacterial activity, induces the formation of dentinal bridge, and has optimal biocompatibility comparable to that of MTA. Due to optimal biocompatibility, it can induce the formation of a cementum-like tissue on surfaces. The main advantages of CEM cement over MTA include its easier handling, lower film thickness, and shorter setting time [ 10 ]. Musale *et al*. [ 11 ] reported that teeth pulpotomized with MTA and Biodentine showed higher success rate than formocresol (92.9% versus 75%) after 12 months. They concluded that Biodentine could serve as a suitable PCA for primary teeth. Acidic Ferric sulfate (FS) is another PCA with a mechanism of action through agglutination of blood proteins. It is mainly introduced as a hemostatic agent that can effectively induce dentinal bridge formation [ 12 ]. 

Saberi *et al*. [ 13 ] evaluated the cytotoxicity of MTA, CEM cement, Biodentine, and octa-calcium phosphate against human gingival fibroblasts, and concluded that the cytotoxicity of MTA, CEM cement, Biodentine, and octa-calcium phosphate was comparable to that of cont-rol group in the first 24-48 hours after exposure. However, MTA and Biodentine showed lower cytotoxicity over time compared with other biomaterials. A review study revealed that teeth treated with MTA had higher clinical success rate (effective inflammatory response and dentinal bridge formation) compared with teeth treated with CHO [ 14 ]. In addition, Dou *et al*. [ 15 ] compared the effects of platelet rich fibrin, concentrated growth factors, iRoot BP, MTA, and CHO on human DPSCs and showed that they were all effective for pulp therapy. Considering the significance of SHEDs in pulpal regeneration and preservation of pulp vitality, this study aimed to assess the cytotoxic effects of four important PCAs namely MTA, Biodentine, CEM cement, and FS on SHEDs. 

## Materials and Method

This study was approved by the Ethics Committee of Zanjan University of Medical Sciences (No. IR.ZUMS. 1397.249). 

### Preparation of materials and specimens

This *in vitro* experimental study was conducted on 8th passage SHEDs obtained from the Rouyan Center of Isfahan, Iran. Dulbecco's Modified Eagle Medium was supplemented with fetal bovine serum to provide nutrients such as albumin, enzymes, and cytokines, and was used in this study. Biomaterials including CEM cement (Yekta Zist Dandan), Biodentine (Septodont), 20% FS gel (Sultradent), and MTA Angelus (Angelus; Londrina, PR, Brazil) were prepared according to the manufacturers’ instructions. 

### Preparation of culture medium

Materials in 2 mm thickness were applied at the bottom of 24-well plates, and were incubated at 37°C and 5% CO2 for 4 hours for setting. Next, 1 mL of Dulbecco’s modified Eagle’s medium was added to the well-plates coated with the biomaterials. The plates containing the culture medium were incubated at 37°C and 5% CO2 for 18 hours. Five different concentrations (1:1, 1:2, 1:4, 1:8, and 1:16) of the culture medium exposed to biomaterials were prepared by serial dilution. 

### MTT assay

This assay is suitable for the measurement of drug sensitivity in established cell lines as well as primary cell [ 16 ]. A cell suspension was prepared for the MTT assay (MTT Cell Growth Assay Kit, Chemicon, Rosemont, IL, USA); 200 µL of the suspension was added to each well of a 96-well plate. To ensure the adequate number of cells in each well, the trypan blue cell viability assay was performed. Next, the cells were incubated in presence of 5% CO2 and 100% humidity for 24 hours to ensure their adherence to the bottom of the wells. Finally, the biomaterial suspension in five different concentrations was added to the cells. The plates were incubated for 24 and 72 hours. After 24 hours, the overlaying culture medium was removed from the first plate, and 200 µL of fresh culture medium was added to each well. Next, 30µL MTT dye was added to each well. The plate was covered with aluminum foil and incubated at 37°C for 4 hours. After this time period, the culture medium was removed, and 100 µL of dimethyl sulfoxide was added to each well plus 10 µL of glycine buffer [ 17 - 18 ].

### Measurement of optical density (OD)

The OD of each well was measured by spectrophotometry using an ELISA Reader (ELX800; BioTek Instruments, Inc., USA) at 570 nm wavelength. The OD for each concentration of each biomaterial was recorded at 24 and 72 hours to find the cell proliferation rate by drawing the respective curves. The percentage of cell viability in wells exposed to the biomaterials compared with the control wells was calculated using the following formula: OD of cells exposed to biomaterials in each well x 100/mean OD of control wells.

### Statistical analysis

The measures of central dispersion and the frequency values were reported, and data were analyzed by one-way ANOVA followed by the Tukey’s post-hoc test in Grap-hPad Prism software, *p*<0.05 was considered significant. 

## Results

Comparison of cell viability in presence of different concentrations of the same biomaterial at a certain time point revealed no significant difference among 1, 0.25, and 0.5 concentrations of Biodentine at 72 hours. However, the cell viability was significantly higher in 0.125 (*p*< 0.05) and 0.063 (*p*< 0.01) concentrations compared with the concentration of 1. In other words, decreasing the concentration significantly increased cell viability (*p*< 0.000). Also, cell viability in 0.5, 0.25, 0.125, and 0.063 concentrations was significantly higher than the control group at 72 hours (*p*< 0.05). No significant difference was noted in cell viability in presence of concentrations of 1, 0.5, and 0.25 of CEM cement, and the difference with the control group was not significant either at 24 hours. However, significant differences were noted between 0.125 (*p*< 0.01) and 0.063 (*p*< 0.001) concentrations with the concentration of 1, indicating an increase in cell viability by a reduction in concentration of CEM cement. In addition, at 72 hours, significant differences existed between 0.5, 0.125, and 0.063 concentrations and maximum concentration, such that by a reduction in concentration, cell viability significantly increased at 72 hours (*p*< 0.0001). 

Cell viability was not significantly different among 1, 0.5, and 0.25 concentrations of FS, and the difference with the control group was not significant either at 72 hours. However, cell viability was significantly higher in 0.125 and 0.063 concentrations compared with the concentration of 1 (*p*< 0.0001). Significant differences were noted between 1, 0.5, 0.25 and 0.125 with minimum concentration (*p*< 0.0001) and also between 0.5 and 0.125 concentrations (*p*< 0.001), indicating an increase in cell viability by a reduction in concentration at 72 hours. 

No significant difference was noted in cell viability in presence of different concentrations of MTA at 24 hours, and the difference with the control group was not significant either. At 72 hours, cell viability in presence of minimum concentration was significantly higher than that in presence of maximum concentration (*p*< 0.0001). 

At 72 hours, cell viability in presence of 0.5, 0.25, 0.125, and 0.063 concentrations of all four biomaterials was higher than that in the control group (*p*< 0.05). 

Comparison of cell viability in presence of similar concentrations of the same biomaterial at 24 and 72 hours showed a significant increase in cell viability and biocompatibility of materials over time (*p*< 0.001). In addition, cell viability was not significantly different in presence of similar concentrations of Biodentine, CEM cement, FS, and MTA at 24 or 72 hours.

**Figure 1:**
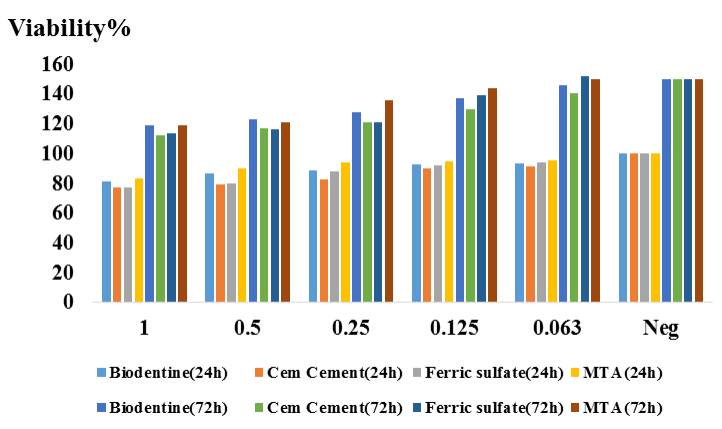
Percentage of cell viability in presence of different concentrations of pulp capping agents (PCAs) at two times; Mineral trioxide aggregate (MTA), calcium enriched mixture (CEM) cement

**Figure 2:**
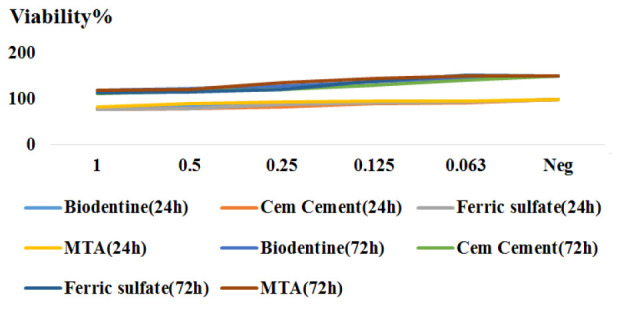
Cell viability in different concentrations of pulp capping agents (PCAs) at two times, Mineral trioxide aggregate (MTA), calcium enriched mixture (CEM) cement

Figures 1-2 and Table 1 show the results of the MTT assay regarding the percentage of cell viability in prese-nce of different concentrations of Biodentine, CEM cement, FS, and MTA and 24 and 72 hours.

**Table 1:** Results of the methyl thiazolyl tetrazolium (MTT) assay

Concentrations	Pulp capping agents (PCAs) materials	Number of cell viability (%)	
		24 hour	72 hour
1	Biodentine	81.33(1.5727)	119(5.291)
	CEM cement	77.33(2.516)	112.33(2.516)
	FS	77.66(0.577)	113.66(4.725)
	MTA	83.33(0.015)	119(0.036)
0.5	Biodentine	86.66(1.527)	123(4.358)
	CEM cement	79.33(2.081)	117.33(2.5160
	FS	80(1)	116.33(5.686)
	MTA	90.33(0.015)	121(0.036)
0.25	Biodentine	88.66(1.154)	127.66(2.516)
	CEM cement	82.66(2.516)	121(3.605)
	FS	88.33(3.214)	121(5.291)
	MTA	94.33(0.020)	136(0.165)
0.125	Biodentine	92.66(2.516)	137(3.605)
	CEM cement	90.33(0.577)	130(1)
	FS	92.33(2.516)	139.67(0.136)
	MTA	94.67(0.005)	144(0.072)
0.063	Biodentine	93.33(1.527)	146.33(4.725)
	CEM cement	91.33(1.527)	141(3)
	FS	94(3.605)	152(3.605)
	MTA	95.33(0.015)	150(0.062)
Primary cellules with without adding PCAs	__.__	100	155

MTA: Mineral trioxide aggregate, CEM: Calcium enriched mixture (cement, ferric sulfate (FS)

## Discussion

It appears that DPSCs are suitable for *in vitro* studies regarding the biological response following exposure to different materials, and enable the assessment of cytotoxicity/ biocompatibility of materials. SHEDs were used in this study due to high potential for proliferation and differentiation into different cell lines. SHEDs were used as an ideal cell line model in several studies [ 19 ]. This study assessed the cytotoxicity of four important PCAs by the MTT assay. The results showed that by a reduction in concentration of Biodentine, CEM cement, and FS, their biocompatibility increased. This finding was probably due to a reduction in concentration of materials and decreased effect of their microparticles in the culture medium on SCs. Jaberiansari *et al*. [ 20 ] compared CEM cement, MTA, and New CEM cement and reported that their biocompatibility increased with a reduction in concentration, which was in agreement with the present results. 

In the present study, Biodentine, CEM cement, FS, and MTA at 1, 0.5, 0.25, 0.125, and 0.063 concentrations were evaluated at 24 and 72 hours regarding their cytotoxic effects on SHEDs. The results showed an increase in cell viability in presence of all concentrations of Biodentine at 24 hours compared with the baseline concentration, which can be due to increased biocompatibility as the result of reduction in concentration of Biodentine. Cell viability significantly increased in presence of 0.125 and 0.063 concentrations at 72 hours (*p*< 0.05). This result was in line with the findings of Collado-González *et al*. [ 2 ] who assessed the cytotoxicity and biocompatibility of pulp capping agents. Zakerzadeh *et al*. [ 21 ] reported similar results as well, although they assessed the cytotoxicity of Biodentine against fibroblasts. Moshary *et al*. [ 22 ] evaluated the cytotoxicity of ProRoot MTA and Endocem, and reported that maximum concentration of Endocem at 24 hours showed the highest cytotoxicity, while the lowest cytotoxicity was related to the minimum concentration of ProRoot MTA at 72 hours. Cell viability increased at both 24 and 72 hours by a reduction in concentration of CEM cement, which was consistent with the findings of Jaberiansari *et al*. [ 20 ] who reported an increase in cell proliferation by a reduction in concentration of CEM cement. In contrast to the study by Jaberiansari *et al*. [ 20 ], who only assessed 1 and 0.5 concentrations, five different concentrations were compared in the present study [ 20 ]. The current results also revealed a reduction in cytotoxicity by a reduction in concentration of FS at both 24 and 72 hours. Nonetheless, Nowakowski *et al*. [ 23 ] evaluated the cytotoxicity of FS in two different concentrations against gingival fibroblasts after 3, 5, and 10 minutes, and 24 hours of exposure, and reported a cytotoxicity up to 50%. Difference between their results and the present findings can be due to the fact that they assessed the cytotoxicity of FS early after exposure while we assessed its cytotoxicity after 24 and 72 hours. 

In the present study, cytotoxicity of materials significantly decreased at 72 hours compared with similar concentrations at 24 hours (*p*< 0.0001). The suggested reasons may be the high leakage of materials early after exposure and reduction of leakage over time, as well as the higher proliferation of SCs in the early hours. Consistent with the present results, another study evaluated the cytotoxicity of PCAs in three different concentrations after 24, 48, and 72 hours, and showed that the cytotoxicity of MTA and Biodentine decreased with time, due to decreased proliferation of SHEDs [ 2 ]. Omidi *et al*. [ 24 ] evaluated cell proliferation, cytokine release, and migration of DPSCs following exposure to Biodentine, CEM cement, MTA, and TheraCal LC using the MTT assay. They used the trans-well migration assay and enzyme-linked immunosorbent assay at 24, 48 and 72 hours, and demonstrated that different concentrations of Biodentine, CEM cement and MTA had no significant difference with each other at different time points, and were not cytotoxic. However, TheraCal LC showed cytotoxicity at all three tested time points. Margunato *et al*. [ 25 ] evaluated the biocompatibility of MTA, Biodentine, and MM-MTA for alveolar bone SCs, and reported that the cytotoxicity of Biodentine and MTA decreased over time (at 14 days, 7 days, 3 days, and 1 day), which was in agreement with the present findings. Another study assessed the cytotoxicity of MTA, CHO, and iRoot BP and demonstrated the formation of dentinal bridge by CHO due to its high pH and creation of an alkaline environment and subsequent cell necrosis. MTA induced the formation of a strong more predictable dentinal bridge. In addition, MTA and iRoot BP had similar efficacy in eliciting an effective pulpal response in pulp therapy [ 26 ]. 

In the present study, comparison of the cytotoxicity of different biomaterials, irrespective of time and concentration, showed that at both 24 and 72 hours, the cytotoxicity of biomaterials was highly similar, with no significant difference. Although MTA was the gold-standard regarding biocompatibility and showed maximum biocompatibility with no significant difference with other tested biomaterials. Birant *et al*. [ 27 ] evaluated the cytotoxicity of ProRoot MTA, Biodentine, and NeoMTA against DPSCs by the annexin-V test at 24, 72, and 168 hours. They found that cell viability in Biodentine group was higher than that in ProRoot MTA and NeoMTA groups, but not significantly. In general, the tested biomaterials had no cytotoxic effects on DPSCs. Dahake *et al*. [ 28 ] assessed the cytotoxicity of Biodentine, MTA, and EMD on SHEDs using the MTT assay and Alizarin red staining at 14 days. They found that EMD was associated with the highest cell viability at 7 days, and maximum calcification at 14 days, although the differences were not statistically significant. They concluded that all three materials could be recommended as optimal PCAs. Collado-González *et al*. [ 2 ] studied the cytotoxicity of PCAs against SHEDs, and reported that Biodentine was more biocompatible than Intermediate Restorative Material (IRM), MTA Angelus, and TheraCal, and had the lowest rate of cytotoxicity. Difference between their results and the present findings may be due to different methodologies since they added the biomaterials to the culture medium 24 hours after their setting, while in the present study, biomaterials were in contact with the culture medium 4 hours after setting, and then different dilutions of the culture medium were prepared and added to the cells. Omidi *et al*. [ 24 ] compared the cytotoxicity of Biodentine, MTA, and TheraCal against dental pulp fibroblasts, and concluded that they were not significantly different regarding cytotoxicity, which was in agreement with the present findings. This finding may be due to the similar structure of Biodentine and MTA to hydroxyapatite, which includes calcium and phosphate. In addition, they can serve as a biocompatible scaffold and provide a suitable matrix for cell proliferation and adhesion. Jaberiansari *et al*. [ 20 ] evaluated the cytotoxicity of several materials against gingival fibroblasts. They used FS in two forms of 15.5% FS solution and 20% FS gel. The MTT assay was performed at 3, 5 and 10 minutes and also after 24 hours. Consistent with the present results, FS had maximum cytotoxicity compared with aluminum sulfate, and aluminum chloride, and 15.5% FS had higher cytotoxicity, which appears to be due to the rheological properties of 15.5% FS solution; whereas, 20% FS gel has lower acidity due to its lower flow ability. Manaspon *et al*. [ 29 ] compared the cytotoxicity of four PCAs (DyCal R, ProRoot R MTA, Biodentine, and TheraCal) on human dental pulp stem cells (hDPs) with the control. This study showed that TheraCal and DyCal R were cytotoxic *in vitro* while ProRoot R MTA and Biodentine demonstrated the better biocompatibility to hDPs. Their results are comparable to previous researches in SHEDs. In contrast, our study demonstrated no significant difference at any time point regarding cytotoxicity of different biomaterials. However, MTA and Biodentine showed maximum biocompatibility in our study similar their study. This could be justified due to the fact that they assessed the dental pulp tissues separated from the extracted permanent teeth, while our study was conducted on mesenchymal stem cells isolated from deciduous teeth. Additionally, they assessed TheraCal a resin based material that set with light cure and it can be one reason for toxicity. 

The main strength of this study was comparison of cell viability in presence of different concentrations of PCAs. Nonetheless, future studies may use other biocompatibility testing assays such as the Comet test, Tunel WST, DNA covalent binding assays, and quantitative real-time polymerase chain reaction. Moreover, assessment of internal factors and cell cycle for evaluation of cell viability, and the biological response of different cell lines in contact with different concentrations of capping agents can provide valuable information and 

help in selection of the best material for pulp capping.

## Conclusion

These results showed comparable cytotoxicity of MTA, Biodentine, CEM cement, and FS, with no significant difference among them. Cytotoxicity decreased over time and by reduction in concentration of biomaterials. Nonetheless, MTA and Biodentine showed maximum biocompatibility followed by FS and CEM cement.
